# Probiotics in milk replacer affect the microbiome of the lung in neonatal dairy calves

**DOI:** 10.3389/fmicb.2023.1298570

**Published:** 2024-01-05

**Authors:** Tara G. McDaneld, Susan D. Eicher, Aaron Dickey, Janice E. Kritchevsky, Keith A. Bryan, Carol G. Chitko-McKown

**Affiliations:** ^1^USDA, ARS, U.S. Meat Animal Research Center, Clay Center, NE, United States; ^2^Livestock Behavior Research Unit, USDA, ARS, West Lafayette, IN, United States; ^3^Department of Veterinary Clinical Sciences, College of Veterinary Medicine, Purdue University, West Lafayette, IN, United States; ^4^Chr. Hansen, Inc., Milwaukee, WI, United States

**Keywords:** dairy cattle, 16S rRNA, lavage, microbiome, probiotic

## Abstract

**Introduction:**

Probiotics have been investigated for their many health benefits and impact on the microbiota of the gut. Recent data have also supported a gut–lung axis regarding the bacterial populations (microbiomes) of the two locations; however, little research has been performed to determine the effects of oral probiotics on the microbiome of the bovine respiratory tract. We hypothesized that probiotic treatment would result in changes in the lung microbiome as measured in lung lavage fluid. Our overall goal was to characterize bacterial populations in the lungs of calves fed probiotics in milk replacer and dry rations from birth to weaning.

**Methods:**

A group of 20 dairy calves was split into two treatment groups: probiotic (TRT; *N* = 10, milk replacer +5 g/d probiotics; Bovamine Dairy, Chr. Hansen, Inc., Milwaukee, WI) and control (CON; *N* = 10, milk replacer only). On day 0, birth weight was obtained, and calves were provided colostrum as per the dairy SOP. On day 2, probiotics were added to the milk replacer of the treated group and then included in their dry ration. Lung lavages were performed on day 52 on five random calves selected from each treatment group. DNA was extracted from lavage fluid, and 16S ribosomal RNA (rRNA) gene hypervariable regions 1–3 were amplified by PCR and sequenced using next-generation sequencing (Illumina MiSeq) for the identification of the bacterial taxa present. Taxa were classified into both operational taxonomic units (OTUs) and amplicon sequence variants (ASVs).

**Results:**

Overall, the evaluation of these samples revealed that the bacterial genera identified in the lung lavage samples of probiotic-fed calves as compared to the control calves were significantly different based on the OTU dataset (*p* < 0.05) and approached significance for the ASV dataset (*p* < 0.06). Additionally, when comparing the diversity of taxa in lung lavage samples to nasal and tonsil samples, taxa diversity of lung samples was significantly lower (*p* < 0.05).

**Discussion:**

In conclusion, analysis of the respiratory microbiome in lung lavage samples after probiotic treatment provides insight into the distribution of bacterial populations in response to oral probiotics and demonstrates that oral probiotics affect more than the gut microbiome.

## Introduction

Feed additives such as probiotics have the potential to impact ruminal mechanisms of action when fed to cattle and, as a result, can influence ruminal fermentation by optimizing fiber degradation and the efficiency of microbial protein synthesis, allowing nutrients to be used more efficiently (DeVries and Chevaux, 2014; Yuan et al., 2015; Dias et al., 2018). Probiotics are also reported to alter the expression of gut epithelial genes promoting barrier function (Petri et al., 2020) and impact inflammatory responses to various stresses in dairy cattle that may allow young calves to resist inflammation-based diseases (Alugongo et al., 2017).

It is recognized that the gastrointestinal tract is the largest immune organ in an animal, and the microbiota of the gut is influenced by the mucosal immune system, and the microbiota of the gut impacts the immune system (Chase, 2018). Lymphocytes of the immune system can also travel between mucosal-associated lymphoid tissues, which include the gastrointestinal tract and the respiratory system, through the “common mucosal immune system” (Chase, 2018). As a result, it has been reported that dysbiosis at either site (gastrointestinal tract and respiratory system) can promote inflammation and dysbiosis at the other site (Vientos-Plotts et al., 2023). Mateer et al. (2015) took this one step further and reported that the respiratory microbiome is influenced by immigration from the gut through potential aspiration, which may impact the microbiota of the lungs. This cross-talk between gut microbiota and lung immunity, known as the gut-lung axis, has been reported in chickens (Saint-Martin et al., 2022), cattle (Chase, 2018), and humans (Rastogi et al., 2022). Recent data have supported a gut–lung axis in microbiome profiles of the two niches, as bacterial taxa previously thought to be predominant in the gut have been found in the lung and vice versa (Budden et al., 2017; Glendinning et al., 2017; Enaud et al., 2020; Narayana et al., 2023). A review by Rastogi et al. (2022) reported that the loss of the gut–lung axis is associated with increased susceptibility to airway disorders, with patients suffering from gut disorders having an increased risk of pulmonary tract disorders.

While oral probiotics have been investigated for their impact on ruminal fermentation, they have also been reported to influence the microbial populations (microbiomes) of the rumen (Pinloche et al., 2013; Azad et al., 2017; Schofield et al., 2018), intestinal tract (Liu et al., 2022), and feces (Fernández-Ciganda et al., 2022; Mansilla et al., 2022). However, little research has been performed to determine the effects of oral probiotics on the microbiome of the bovine respiratory tract. This study aimed to characterize the bacterial populations present in the lungs of pre-weaning dairy calves after treatment with oral probiotics. As a result, evaluation of the animal’s respiratory microbiome in response to oral probiotics may clarify the impact of probiotics on the bacteria population in the lung of pre-weaning dairy calves.

## Materials and methods

### Animal populations

Data were collected in 2018 at the Purdue University Dairy Teaching and Research Unit. All animal use was approved by the Purdue Animal Care and Use Committee (#1803001701). Twenty Holstein calves were placed in the study after meeting the criteria of birth weight between 32 and 50 kg and having a plasma protein value equal to or greater than 5.5 g/dL measured by Brix refractometry between 24 and 48 h after birth. Calves were given 1 L of superior-grade colostrum within 12 h of birth and again within 24 h of birth. Calves were then fed 2 L of 24/20 milk replacer (Milk Specialties Global, Eden Prairie, MN) and divided into two equal feedings per day. Beginning on day 2 of life, calves were moved to individual hutches and assigned to probiotic (TRT) or control (CON) treatments of milk replacer. Probiotics (Bovamine Dairy, Chr. Hansen, Inc., Milwaukee, WI) were delivered into each bottle (2.5 g/bottle, two bottles a day) at feeding. Probiotics were kept refrigerated after being aliquoted for each feeding until used. The probiotics consisted of lactose, sodium silico aluminate, dried *Propionibacterium freudenreichii* fermentation product, and dried *Lactobacillus animalis* fermentation product (1.5 × 10^9^ CFU/g). This commercially available direct-fed microbial was selected as it is one of the most widely used combinations of bacterial strains in beef and dairy cattle diets featuring lactate-producing and lactate-utilizing probiotic bacteria that have action in the rumen (Ban and Guan, 2021). Direct-fed microbials have been shown to improve health outcomes in cattle (McAllister et al., 2011), and feeding direct-fed microbials may improve the immune responses of cattle (McAllister et al., 2011; Buntyn et al., 2016). Calves were weaned (step-down) on day 42. Basically, one milk feeding was discontinued at day 42, and the second was discontinued based on dry feed consumption (after the consumption of feed at approximately 1.5 kg/d). Probiotics were also included in the dry feed at a targeted intake of approximately 5 g/d from day 7 until after weaning was completed. Dry feed was available from days 7 to 52. Calves were weighed individually at birth and then weekly until day 49. Calves were assigned to a Tuesday or Friday weekly weigh day (whichever was closest to their birth date). Calves were scored daily for fecal scores (Eicher-Pruiett et al., 1992), ocular and nasal discharge, ear orientation, and overall clinical score. During the duration of the study, none of the calves were diagnosed or treated for respiratory disease.

### Sample collection

On day 52, lung lavages were performed on five calves randomly selected from the 10 calves in each treatment group (TRT, *N* = 5 and CON, *N* = 5). Prior to the lung lavage, cetacaine was sprayed into the left nostril of the calf after moving them to the barn near where they were housed. While calves were gently restrained by two people, the end of a bovine bronchoscope was sprayed with cetacaine and inserted through the nostril and into the trachea. A flexible 10 French catheter (36″ in length) was inserted through the bronchoscope, and 120 mL of sterile saline at 37°C was infused into the lungs using 60-mL syringes. Immediately after the 120-mL infusion, negative pressure was applied to aspirate the fluid back through the catheter and into a sterile 50-mL endotoxin-free centrifuge tube. The process was repeated to obtain a second sample if necessary to obtain a total of 50 mL of lavage fluid. Samples were placed on ice and then stored at −80°C. These samples were used for the evaluation of the microbiome through amplification and sequencing of the 16S rRNA gene.

As a separate part of the study, nasal and tonsil swabs were collected from the upper respiratory tract of all calves on days 0, 7, 14, 21, 28, 35, 42, and 49 of the study. For nasal sampling, the nose of the animal was wiped clean with a single-use towel if fecal material was present. One unguarded 15.24 cm nasal swab was then gently inserted into each nasal cavity at an approximate depth of at least 14 cm. The nasal swabs were then rotated and removed. After the collection of the sample, the swabs were placed in buffered peptone water with 12% glycerol for subsequent bacterial taxa evaluation. For tonsil sampling, the calves’ mouth was held open, and the tongue was held to the side by hand. A swab was inserted and moved back and forth against the left tonsil, and this was repeated for the right tonsil. The swab was then removed from the calves’ mouth and placed in buffered peptone water with 12% glycerol for subsequent bacterial taxa evaluation. For the data presented herein, nasal and tonsil samples on day 49 from the 20 calves in the study (TRT, *N* = 10 and CON, *N* = 10) were used for the evaluation of bacterial taxa diversity when compared to the lavage samples on day 52, as day 49 was the closest timepoint to day 52.

### DNA extraction for 16S rRNA sequencing for bacterial taxa evaluation

Total DNA was extracted from day 52 lavage samples of all 10 calves sampled (TRT, *N* = 5 and CON, *N* = 5) and from day 49 nasal and day 49 tonsil samples of all 20 calves sampled (TRT, *N* = 10 and CON, *N* = 10) using a commercial kit (PowerSoil DNA kit; Qiagen, Germantown, MD), and initial DNA quantity was evaluated with a DNA spectrophotometer (DeNovix DS-11 FX Series; Wilmington, DE). PCR-grade water was used as the negative control and processed with the other samples in the DNA extraction process to evaluate contamination in the kit reagents. Amplification of the 16S rRNA gene V1–V3 hypervariable region was then completed for each DNA sample using standard PCR (AccuPrime, Invitrogen, Carlsbad, CA) and primers with index sequences as previously described that amplify hypervariable regions 1–3 of the 16S rRNA gene (Myer et al., 2015). The quality and quantity of the resulting 16S rRNA gene amplification were checked on the fragment analyzer (Advanced Analytical, Ankeny, IA). By using indexed primers to amplify the 16S rRNA gene, individual samples were pooled into a single sequencing run and then sequenced utilizing the MiSeq Illumina Sequencer (Illumina, San Diego, CA) with a MiSeq Reagent Kit v3 to generate 2 × 300 paired-end reads. Samples that did not pass the initial quality score cutoff of Q20 > 75% for sequence reads were run in a second sequencing run. Approximately 300,000–560,000 reads were further evaluated for each sample in the pool.

### Data analysis of 16S rRNA data for the identification of operational taxonomic units

The paired-end data files for each DNA sample were downloaded from the MiSeq Illumina Sequencer and processed for the identification of operational taxonomic units (OTUs) and amplicon sequence variants (ASVs) for the comparison of the two methods for classifying bacterial taxa. For the identification of OTUs, the MICCA computing environment for sequence data was used (Albanese et al., 2015). For the lavage samples, OTUs were identified from high-quality sequences based on a 97% identity cutoff. Taxonomic assignment for the identified OTUs was determined using the Naïve Bayesian assignment based on composition similarity (Garcia-Etxebarria et al., 2014) against the Silva database (version SILVA database 138) (Quast et al., 2013). Data for all lavage samples and the negative control were then evaluated for common contaminants (Salter et al., 2014) that may have originated from contaminated reagents or consumables during the DNA extraction. If bacterial genera of common contaminants were identified in the dataset, the second swab collected from the animal was extracted for DNA and subsequent 16S rRNA gene amplification. No contaminants were detected in the samples. Data are presented as a relative abundance (%) of each bacterial genus, phylum, and family in the lavage sample. Depending on the sample, 3–95% of the OTUs could not be classified to the genus level and are identified as unclassified and grouped with the less abundant OTUs as others. The OTUs of TRT calves were compared to CON calves using Phyloseq in R (Version 4.2.1) (R Core Team, 2019). Bacterial genera that were greater than 0.5% relative abundance in more than two samples are reported, and the remaining genera of low abundance are grouped and identified as others (Supplementary Figure S1). Information about the top phylum and family are also reported (Supplementary Figures S2A, S3A, respectively). Beta and alpha diversities were calculated by PERMANOVA and pairwise comparison using the Wilcoxon rank sum test, respectively, in R.

### Data analysis of 16S rRNA data for the identification of amplicon sequence variants

**Figure 1:**
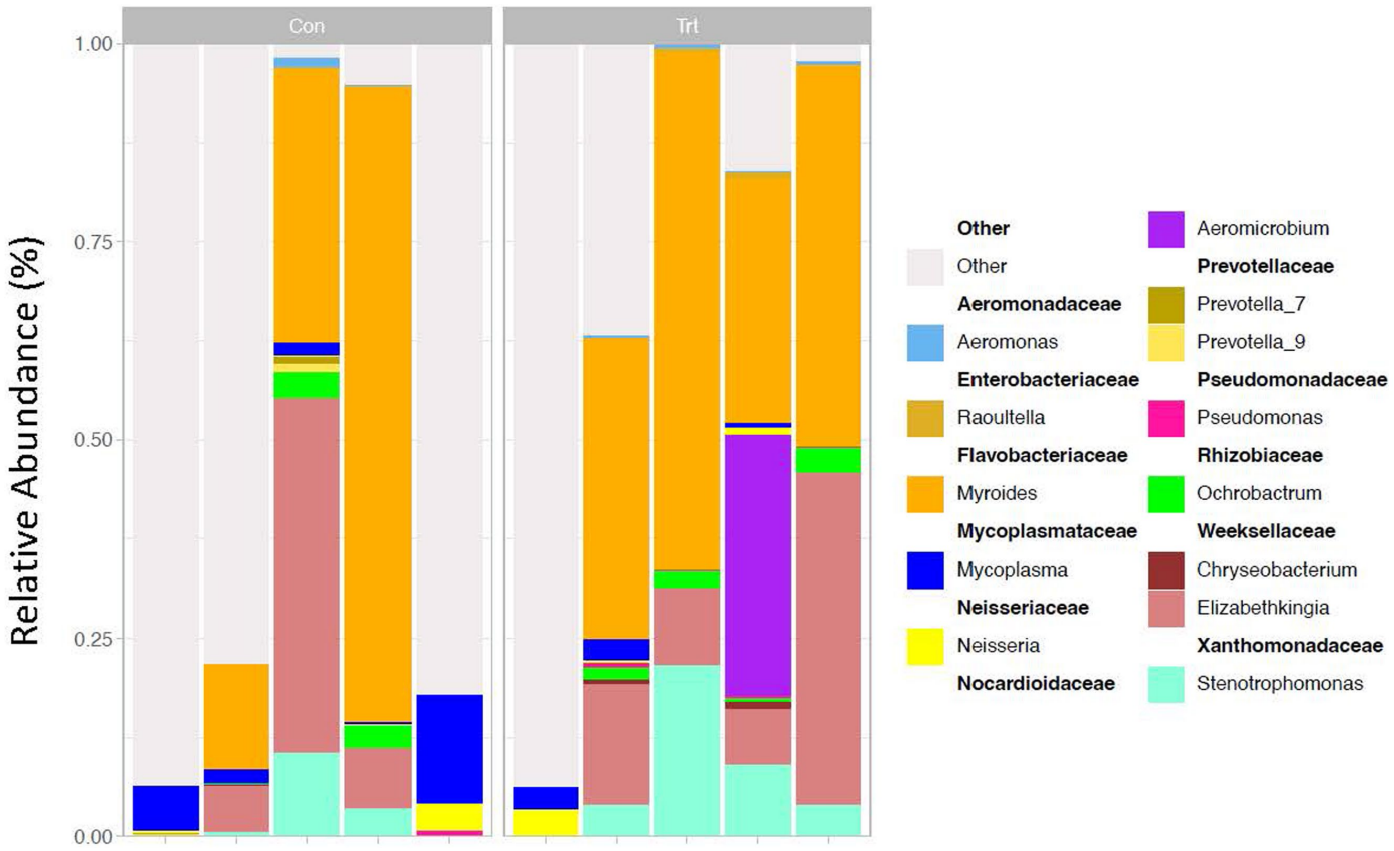
16S rRNA profiles of bacterial genera (taxa classified into ASV) of lung lavage samples from calves provided oral probiotic (TRT) or control calves (CON). 16S rRNA profiles (relative abundance %) were evaluated in probiotic (TRT; *N* = 5) and control (CON; *N* = 5, milk replacer) calves. The genera are presented in the legend as nestled within their corresponding families. Data present the most abundant genera in the lung microbiota. Any ASV that could not be classified to the genus level is grouped as unclassified with the remaining genera of low abundance and identified as other.

For the identification of ASVs, the DADA2 R package for sequence data was used (Callahan et al., 2016). For the paired-end data files, trimming, filtering, and merging were completed with DADA2. Taxonomic classification (Wang et al., 2007) of the merged sequences was performed with DADA2 using the SILVA 138 database. Similar to the methods used for the OTU dataset, the ASVs of TRT calves were compared to CON calves using Phyloseq in R. Bacterial genera that were greater than 0.5% relative abundance in more than two samples are reported, and remaining genera of low abundance are grouped and identified as other with the ASVs that could not be classified to the genus level (Figure 1). Information about the top phylum and family are also reported (Supplementary Figures S2B, S3B, respectively). Beta and alpha diversities were calculated by PERMANOVA and pairwise comparison using the Wilcoxon rank sum test, respectively, in R.

### Data analysis for Venn diagram and functional analysis

For the identification of ASV that were common between the CON and TRT groups of calves, a Venn diagram was constructed using the Russel88/MicEco package with the default settings. Prediction of microbiome function pathway abundances of the classified ASV and samples for each treatment were also performed using PICRUSt 2.0 (Douglas et al., 2020).

## Results

### Alpha and beta diversities

**Figure 2:**
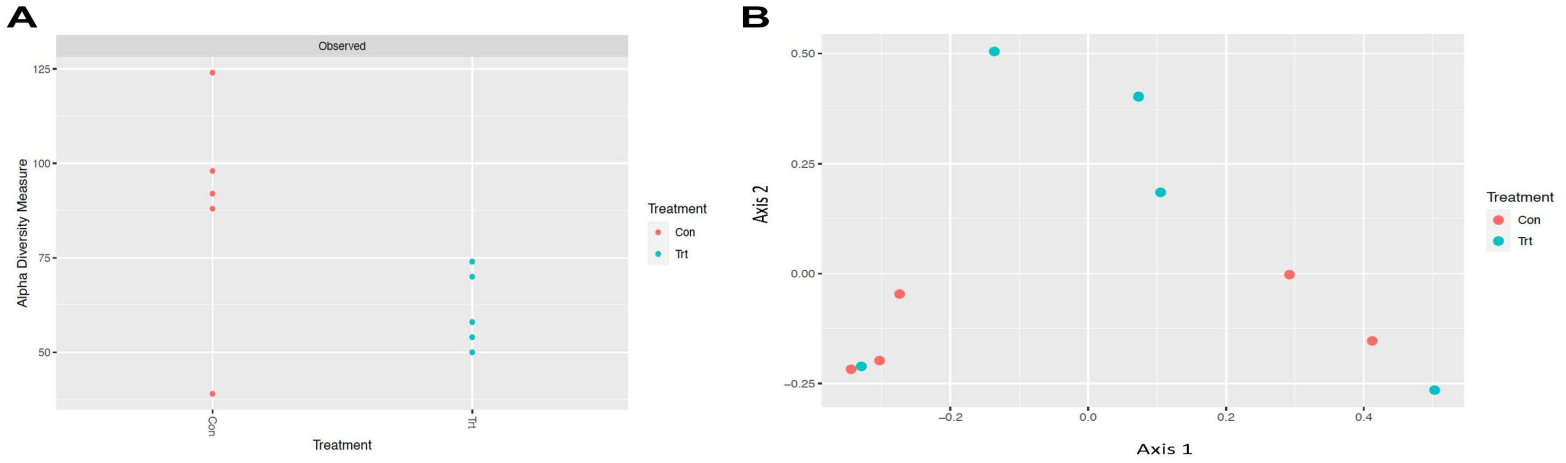
Alpha and beta diversities for bacterial genera (taxa classified into ASV) of lung lavage samples from calves provided oral probiotic (TRT) or control calves (CON). **(A)** Alpha diversity (variation within calf lung lavage samples; *p* = 0.15) for bacterial genera of lavage samples from calves provided oral probiotic (TRT) or control calves (CON). **(B)** Beta diversity (variation across oral probiotic treatment; *p* = 0.10) for bacterial genera of lavage samples from calves provided oral probiotic (TRT) or control calves (CON).

**Figure 3:**
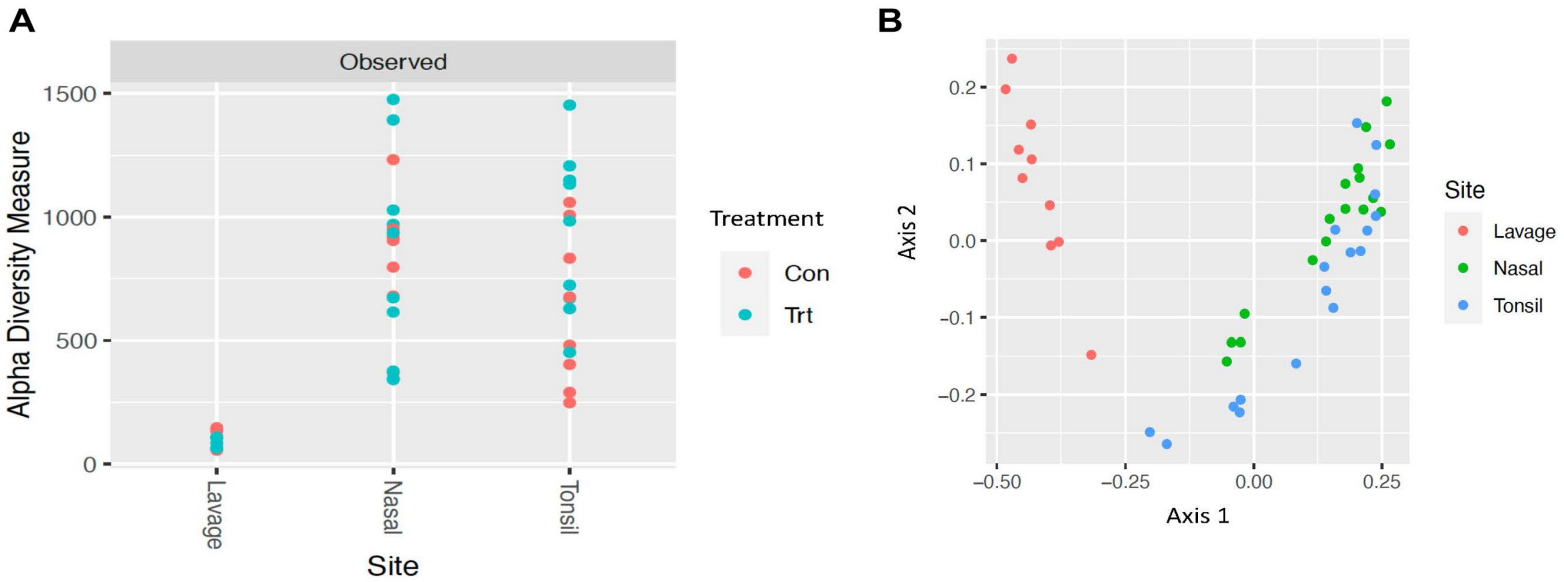
Alpha and beta diversities for bacterial genera of lavage, nasal, and tonsil samples from calves provided oral probiotic (TRT) or control calves (CON). **(A)** Alpha diversity (variation within sample; *p* < 0.05) for bacterial genera of lavage, nasal, and tonsil samples from calves provided oral probiotic (TRT) or control calves (CON). **(B)** Beta diversity (variation across sample site; lavage, nasal, or tonsil; *p* < 0.05) for bacterial genera of lavage, nasal, and tonsil samples for calves provided oral probiotic (TRT) or control calves (CON).

To identify bacterial taxa diversity within each sample of each treatment and across treatments of the lung lavage samples, alpha and beta diversities were evaluated. For both the OTU and ASV datasets, there was no significant difference in alpha diversity (variation within calf lung lavage samples; OTU, *p* = 0.14 and ASV, *p* = 0.15; Supplementary Figure S4A and Figure 2A, respectively) or beta diversity (variation across oral probiotic treatment; OTU, *p* = 0.10 and ASV, *p* = 0.10; Supplementary Figure S4B and Figure 2B, respectively) of the lung lavage samples from TRT and CON calves. However, when comparing the diversity of bacterial profiles of the lung lavage samples at day 52 to nasal and tonsil samples at day 49 (day 49 samples were the closest timepoint to day 52 lavage samples) based on the OTU dataset, alpha diversity of the lung lavage samples was significantly lower (variation within calf samples at each sample sites: lavage, nasal, and tonsil; *p* < 0.05; Figure 3A). Beta diversity (variation across sample site: lavage, nasal, and tonsil) of the bacterial profiles based on the OTU dataset also revealed a separation of the lung lavage sample group versus the nasal and tonsil sample groups (*p* < 0.05; Figure 3B).

### Bacterial taxa evaluation

Evaluation of the bacterial taxa generated through 16S rRNA sequencing identified that the top five taxa based on OTU and ASV classifications of all lung lavage samples were similar and included *Myroides, Elizabethkingia, Stenotrophomonas, Mycoplasma*, and *Aeromicrobium* at the genus level (taxa reported for OTU in Supplementary Figure S1 and taxa reported for ASV in Figure 1). Samples were also evaluated for contamination of the probiotic used in the study. Neither *Propionibacterium freudenreichii* nor *Lactobacillus animalis* were identified, indicating that our samples were not contaminated with the probiotic provided and that the bacteria in the probiotic did not contribute to the lung microbiome in the calves.

For the phylum and family level classification, a greater number of ASVs (>95% for all samples) were classified at the phylum and family level compared to OTUs (Supplementary Figures S2, S3, respectively). For OTU at the phylum and family level, three of the lung lavage samples had less than 25% of the taxa classified, compared to greater than 95% taxa classification for the ASVs.

For the OTU dataset, the genus *Myroides* was identified to be significantly different in relative abundance when comparing the TRT and CON calves (*p* = 0.05). Additionally, the genera *Prevotella 9, Lachnospiraceae, Veillonella, Bosea, Sphingobacterium, Porphyromonas, Finegoldia*, and *Massilia* (*p* = 0.05), and *Flavobacteriaceae* at the family level (*p* = 0.05) were identified to be significantly different between TRT and CON calves for the OTU dataset. With the exception of *Myroides* and *Prevotella 9*, the other genera reported herein for the OTU dataset were of low abundance (<5%) and not present in all the lung lavage samples.

When evaluating the ASV dataset, Prevotella, Dietzia, Oribacterium, Enterobacter, Corynebacterium, Raoultella, Staphylococcus, Lysobacter, Fastidiosipila, Alloprevotella, and Flavobacterium at the genus level and Weeksellaceae and Muribaculaceae at the family level were identified as significantly different between TRT and CON calves (*p* = 0.06). Similar to the OTU dataset, several of the genera identified to be different between the treatments were of low abundance (<5%).

### Venn diagram and functional analysis

**Figure 4:**
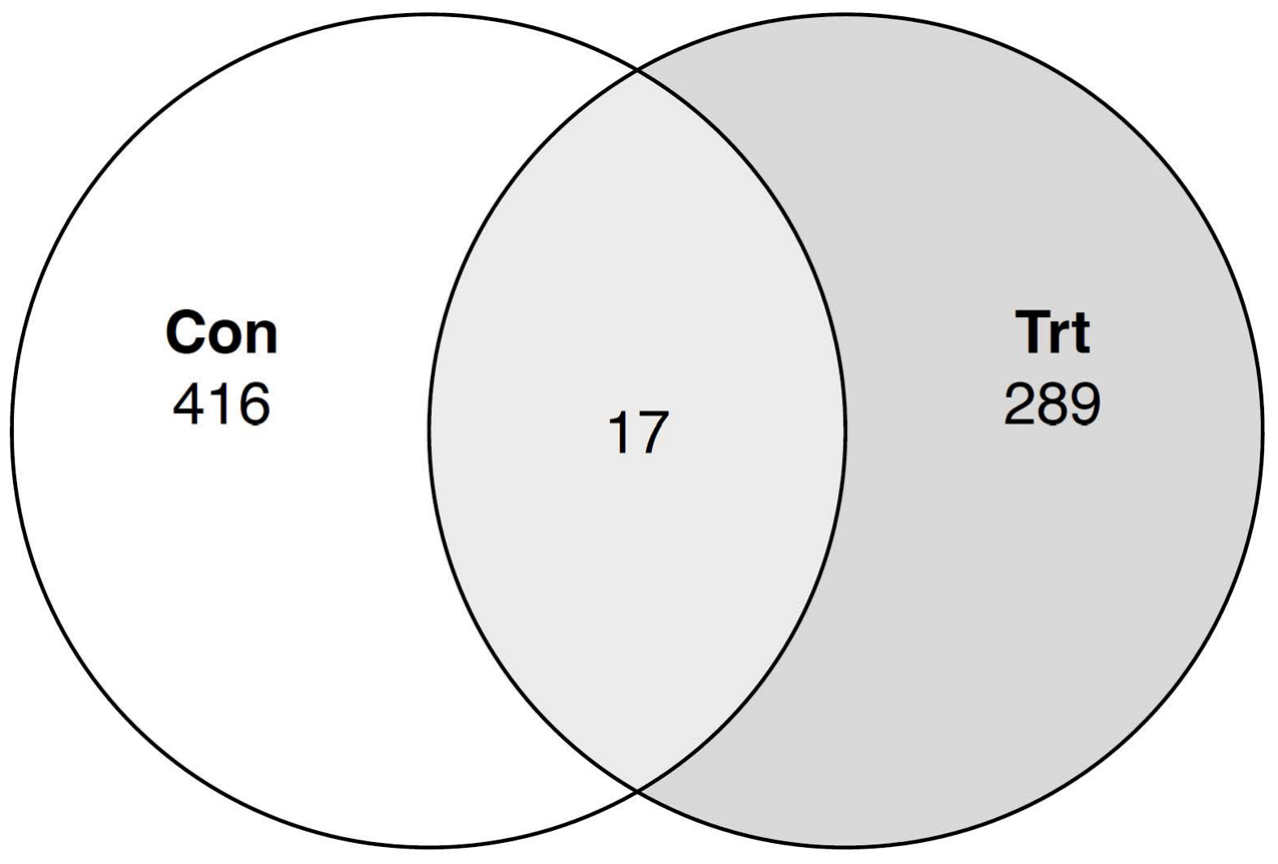
Venn diagram for common bacterial taxa in lung lavage samples from calves provided oral probiotic (TRT) or control calves (CON). Taxonomy classification at the genus level was evaluated between calves provided oral probiotic (TRT; *N* = 5) and control calves (CON; *N* = 5) to identify the common bacterial taxa between the two treatment groups.

A Venn diagram was constructed, and a prediction of the microbiome function pathway abundance of the samples for each treatment was conducted for the ASV dataset. For the Venn diagram, 17 bacterial taxa at the genus level were identified to be common between TRT and CON calves, while the TRT and CON calves had 289 and 416 unique taxa, respectively (Figure 4). Functional prediction identified pathways for each individual sample within each treatment and each individual ASV (Supplementary Tables S1, S2, respectively). No predicted pathways were identified to be significantly different between the treatment groups (*p >* 0.05).

## Discussion

The lung is not a sterile environment, and bacterial species found within may be commensal or pathogenic (Huffnagle et al., 2017; McMullen et al., 2020a; Chai et al., 2022). The bacterial population of the lung has been reported to be influenced by the microbiome of the gut, also referred to as the gut–lung axis. Therefore, this study was completed to identify the bacterial taxa present in the lungs of dairy calves after oral probiotic treatment. Differences among bacterial profiles of lung lavages were determined by comparing calves fed the probiotic (TRT) versus calves not fed the probiotic (CON) during the pre-weaning period. Additionally, for the data presented herein, two common methods were utilized for the classification of bacterial taxa, including OTU and ASV, to compare the classification methods and determine which method was better suited for the classification of taxa for the lung lavage samples.

### Alpha and beta diversities

Alpha and beta diversities of the bacterial profiles were evaluated for the lung lavage samples to identify bacterial taxa diversity within lung lavage samples and across each treatment, respectively. Overall, there was no difference in bacterial taxa diversity within calf lung lavage samples (alpha diversity) or across oral probiotic treatment (beta diversity) of the lung lavage samples from TRT and CON calves. This demonstrates that the treatment of oral probiotics did not significantly influence overall bacterial taxa diversity within each lung lavage sample or across samples of the TRT and CON calves. However, upon comparison of the diversity of bacterial profiles of the lung lavage samples at day 52 to nasal and tonsil samples that were collected from the same calves 3 days earlier, variation within calf samples at each sample site (lavage, nasal, and tonsil; alpha diversity) and across sample sites (beta diversity) was significantly different for the lung lavage samples versus the nasal and tonsil samples regardless of probiotic treatment. These data indicate that the bacterial profiles of the lung lavage samples are significantly different in taxa diversity when compared to the nasal and tonsil samples. While lower alpha diversity is typically associated with disease, previous research has reported a similar lower diversity of the lung compared to other respiratory tract locations of post-weaned beef calves (Nicola et al., 2017) and feedlot cattle (McMullen et al., 2020a; Li et al., 2022). This suggests that the lower diversity of the lung lavage samples, as reported in the data presented herein, is not associated with disease. This is also supported by the fact that none of the calves sampled in the study showed signs of disease or were treated for respiratory disease for the duration of the study.

### Bacterial taxa evaluation

When evaluating the bacterial profiles of the lung lavage samples through 16S rRNA sequencing (expressed as a relative abundance of the bacterial genus in the sample), the top five taxa for both OTU and ASV classifications of all lung lavage samples were similar and included *Myroides, Elizabethkingia, Stenotrophomonas, Mycoplasma*, and *Aeromicrobium* at the genus level. Of these taxa, *Mycoplasma* and *Myroides* have been previously reported to be present in the respiratory tract of pre-weaned calves or feedlot cattle (McDaneld et al., 2019; McMullen et al., 2020a,b; Raabis et al., 2021; Centeno-Martinez et al., 2023). Of the remaining top five genera identified*, Elizabethkingia* and *Stenotrophomonas* have been identified in the respiratory tract of piglets and humans (Hu et al., 2017; Zhang et al., 2020; Menetrey et al., 2021; Chen et al., 2022), but current literature has not reported the genus *Aeromicrobium* as a predominant bacterial taxa present in the respiratory tract. These data suggest that additional research needs to be completed on these bacterial taxa to elucidate the role of these genera in the lungs of cattle.

For the lung lavage dataset, both processes of taxa classification (OTU and ASV) identified the same predominant taxa at the genus level; however, a greater number of ASVs (>95% for all samples) were classified at the phylum and family level compared to OTUs. For example, three of the lung lavage samples had less than 25% of the taxa classified for the OTU dataset compared to greater than 95% taxa classification for the ASVs at the phylum and family level. These data suggest that taxa classification using ASVs is a more robust tool for this lung lavage dataset and may be more informative when evaluating data at higher taxonomic levels.

While the most abundant taxa present in the lung lavage samples did not differ between the OTU and ASV classifications, taxa identified to be significantly different between the lung lavage samples of the TRT and CON calves did differ when comparing the results of the OTU and ASV datasets at the genus level. Of the taxa identified to be of high relative abundance in the lung lavage samples for the OTU dataset, the genera *Myroides* and *Prevotella 9* were identified to be significantly different in relative abundance between lung lavage samples of the TRT and CON calves. Other genera were identified to be significantly different between TRT and CON calves for the OTU dataset; however, these genera reported herein for the OTU dataset were of low abundance (<5%) and not present in all the lung lavage samples evaluated. While the relative abundance of these taxa is low in the calves evaluated, previous literature has identified several of these taxa, including *Lachnospiraceae, Veillonella, Bosea, Sphingobacterium, Porphyromonas, Finegoldia*, and *Massilia* in the microbiome of the respiratory tract of pre-weaned calves (Raabis et al., 2021), feedlot cattle (McMullen et al., 2020a,b), piglets (Zhang et al., 2020), and humans (Hu et al., 2017; Menetrey et al., 2021; Chen et al., 2022).

In comparison, for the ASV dataset, bacterial taxa identified to have different abundances between the TRT and CON calves included *Prevotella, Dietzia, Oribacterium, Enterobacter, Corynebacterium, Raoultella, Staphylococcus, Lysobacter, Fastidiosipila, Alloprevotella*, and *Flavobacterium* at the genus level and *Weeksellaceae* and *Muribaculaceae* at the family level (*p =* 0.06). A *p-value* of 0.06 was used for the ASV data, as no ASV was identified to be significant at a *p*-value of 0.05. Therefore, we used a *p*-value of 0.06 to identify the ASV that was approaching significance and compare this dataset to the OTU dataset. While this set of taxa for the ASV dataset, with the exception of *Prevotella*, is different from the taxa previously identified to be significantly different in abundance between treatments for the OTU dataset, it is of important to note that the literature has only identified the genera *Corynebacterium* and *Staphylococcus* in the respiratory tract of cattle (Zeineldin et al., 2017; Choudhary et al., 2019; Hashem et al., 2022). However, several of these taxa have previously been identified in the microbiome of the respiratory tract of humans (Kim et al., 2013; Lu et al., 2017; Tsay et al., 2018; Chen et al., 2023; Najafi et al., 2023). Similar to the OTU dataset, several of the genera identified to be different between the treatments were of low abundance (<5%). Further research is warranted on a larger set of lung lavage samples to determine the role that these less abundant taxa may play in the lungs of cattle during probiotic treatment.

For the taxa identified to be significantly different in relative abundance in the lung lavage samples for the OTU and ASV datasets, *Myroides, Prevotella, Dietzia, Enterobacter*, *Raoultella*, and *Alloprevotella* have all been previously reported to be identified in the microbiome of the lower respiratory tract and the gut of cattle and humans (Glendinning et al., 2017; Pan et al., 2020; Hérivaux et al., 2022; Narayana et al., 2023). Evaluation of the animal’s bacterial taxa in the respiratory tract in response to oral probiotics is of importance as recent data have supported a gut-lung axis regarding the microbiomes of the two locations as bacterial taxa previously thought to be predominant in the gut have been found in the lung and vice versa (Budden et al., 2017; Glendinning et al., 2017; Enaud et al., 2020; Narayana et al., 2023). It has also been suggested that the microbiome of one location may have profound effects on the microbiome of another location. However, additional research needs to be completed to determine the role of these specific bacterial taxa reported herein in response to probiotics.

### Venn diagram and functional analysis

To further evaluate the taxa present in the TRT and CON calves, common bacterial taxa between the two treatment groups were determined. A total of 17 bacterial taxa at the genus level were identified to be common between the TRT and CON groups of calves. This low number of common taxa was not unexpected, as a majority of the taxa in the calves were of low abundance (<0.5%) and most of the low abundant taxa were not present in all the lung lavage samples. Functional analysis predicted pathways for each sample within each treatment group, and each individual classified ASV. For the pathways identified across all treatment groups, there was no significant difference for probiotic treatment; however, the predicted pathways for each classified ASV allow one to identify pathways that the differentially abundant ASV may have a role in.

## Conclusion

Overall, we were able to demonstrate through next-generation sequencing that the composition of the core bacterial taxa of the lung lavage samples was dominated by *Myroides, Elizabethkingia, Stenotrophomonas, Mycoplasma*, and *Aeromicrobium* at the genus level for both the OTU and ASV datasets. While these were the predominant bacterial agents in all samples evaluated for the data presented herein, *Myroides* was the only predominant bacterial agent identified to be significantly different for probiotic treatment for the OTU dataset, with greater abundance in the calves fed the probiotic. Elucidating the role of these genera in the lungs of cattle is of importance, as several of the taxa identified have not been previously reported in the respiratory tract of cattle. Furthermore, evaluation of the animals’ bacterial populations in the respiratory tract in response to probiotics will improve our understanding of the role of probiotics outside of the rumen. One limitation of this study was the small sample size of five calves per treatment, but these findings provide a direction for future research to explore the impact of oral probiotics on the bacterial profiles of the lungs.

## Data availability statement

The datasets presented in this study can be found in online repositories. The names of the repository/repositories and accession number(s) can be found at: https://www.ncbi.nlm.nih.gov/, the temporary Submission ID is SUB13848781 and BioProject is PRJNA1018539. BioSample accessions: SAMN37441892, SAMN37441893, SAMN37441894, SAMN37441895, SAMN37441896, SAMN37441897, SAMN37441898, SAMN37441899, SAMN37441900, SAMN37441901 will be released 2024-01-31 or with the release of linked data, whichever is first.

## Ethics statement

The animal study was approved by the Purdue Animal Care and Use Committee (#1803001701). This study was supported by the USDA funding CRIS #3040-32000-036-00D, #3040-31000-104-000D, and #5020-32000-013-000-D.

## Author contributions

TM: Formal analysis, Writing – original draft. SE: Conceptualization, Data curation, Investigation, Project administration, Writing – review & editing. AD: Formal analysis, Writing – review & editing. JK: Conceptualization, Data curation, Writing – review & editing. KB: Conceptualization, Resources, Writing – review & editing. CC-M: Conceptualization, Investigation, Writing – review & editing.
