# Supplementary figures and images for: Probiotics in milk replacer affect the microbiome of the lung in neonatal dairy calves
Source: Front Microbiol. 2024 Jan 5;14:1298570. doi: 10.3389/fmicb.2023.1298570 (PMC10797021; doi:10.3389/fmicb.2023.1298570)

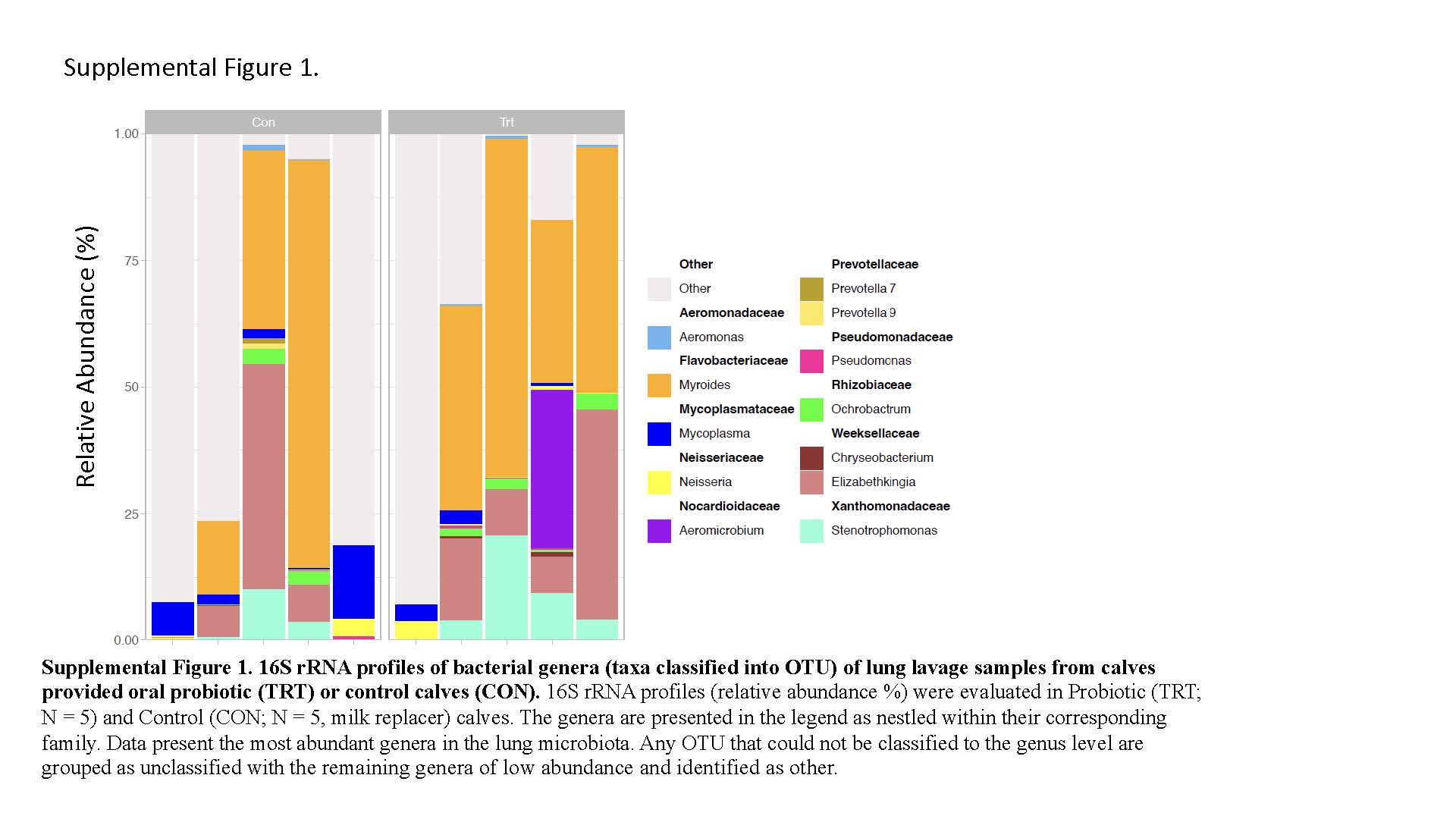

Supplement: Supplementary file 3 [file Image_1.jpg]

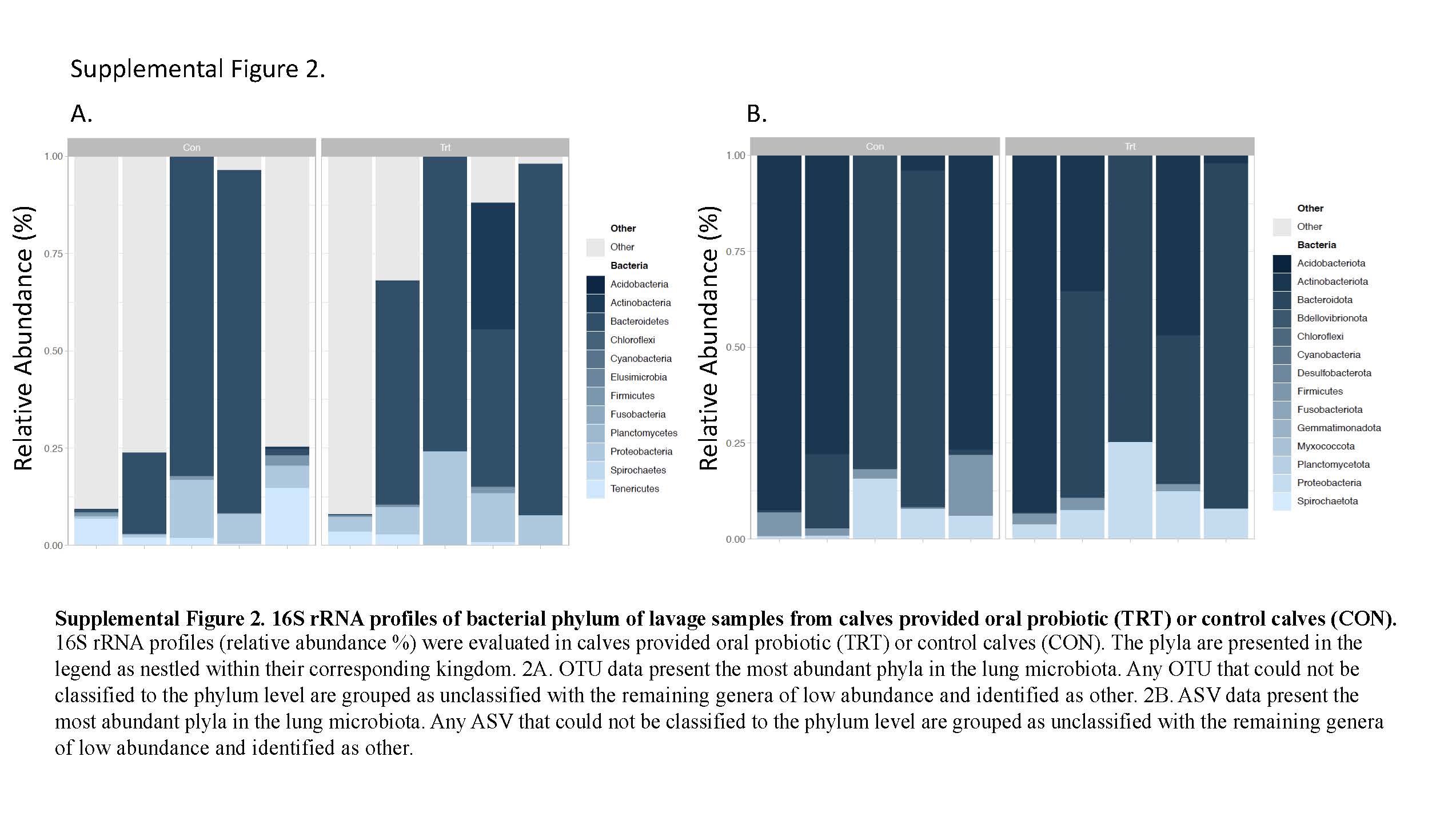

Supplement: Supplementary file 4 [file Image_2.jpg]

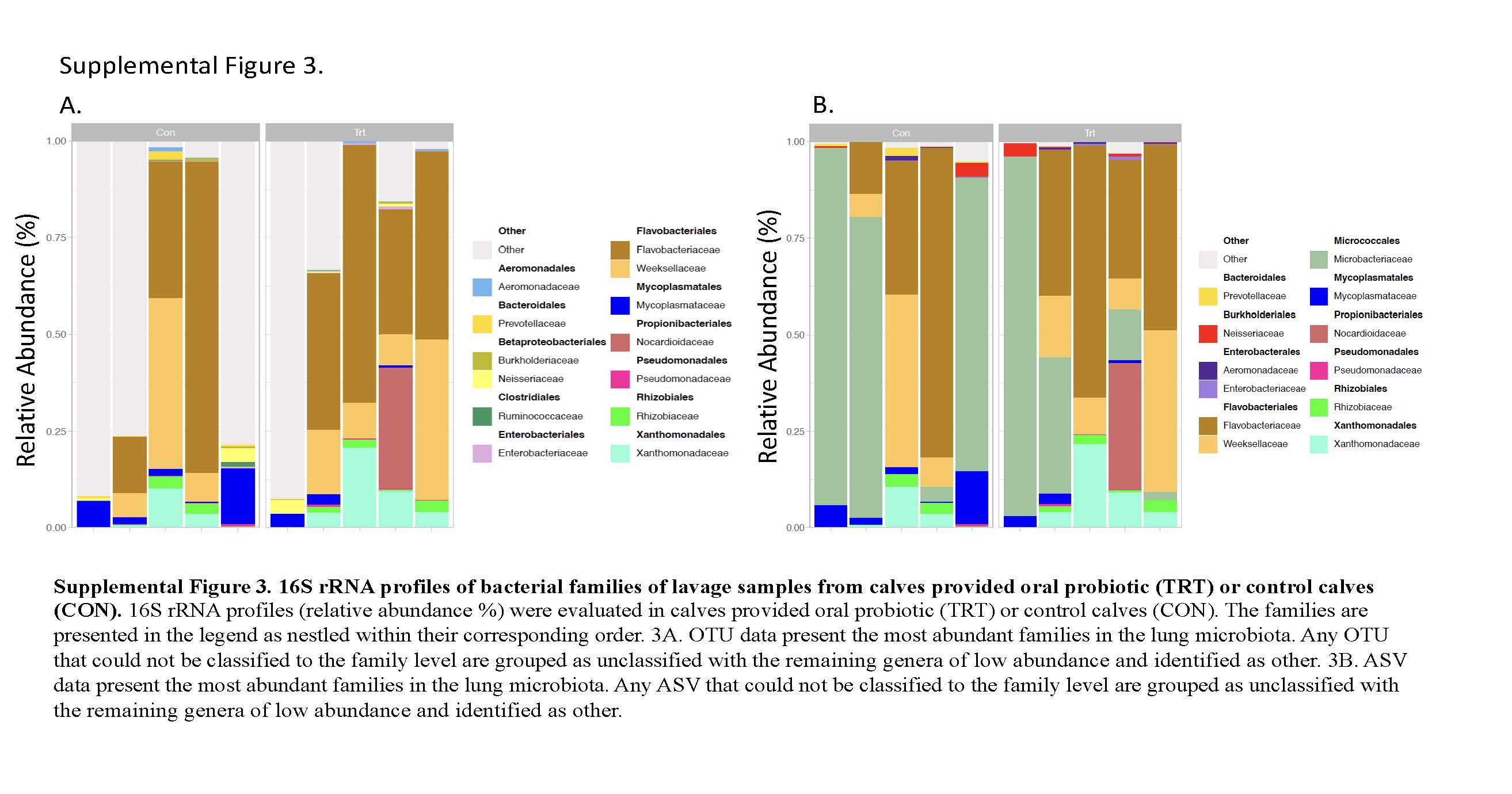

Supplement: Supplementary file 5 [file Image_3.jpg]

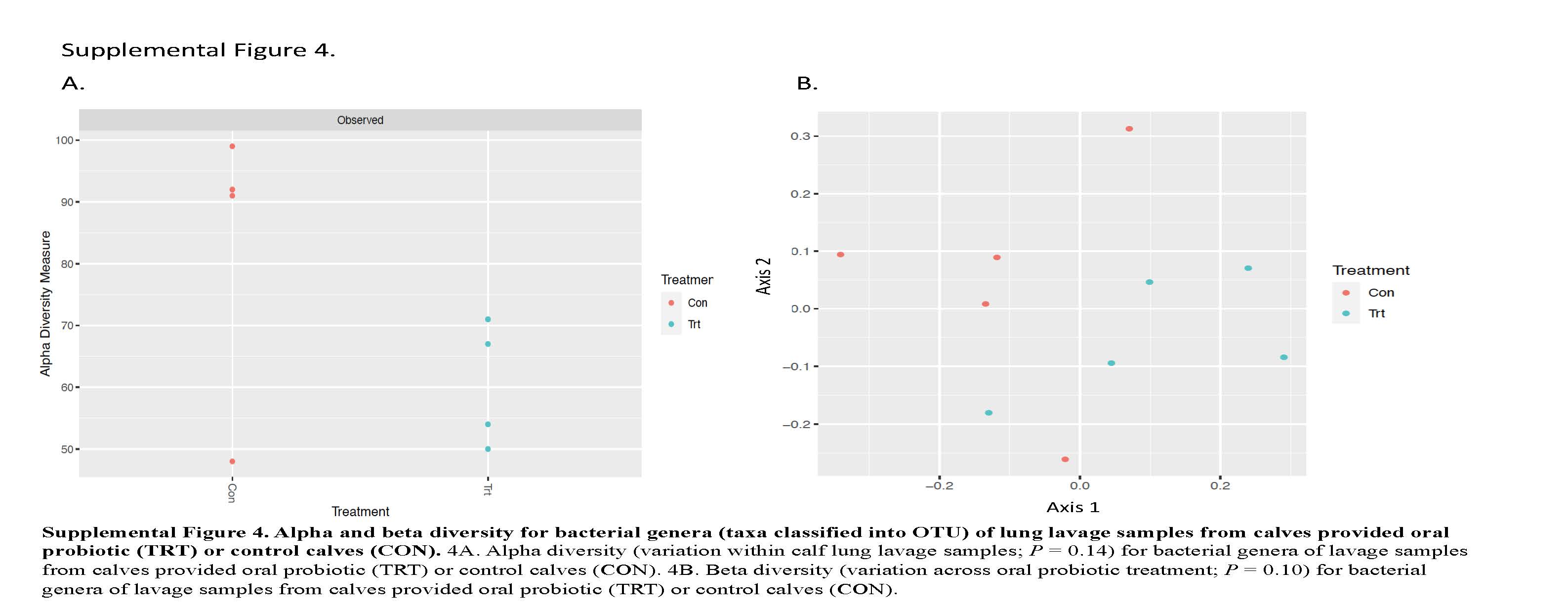

Supplement: Supplementary file 6 [file Image_4.jpg]
